# MEASURING AND IMPLEMENTING PERSON-CENTERED, RESIDENTIAL LONG-TERM CARE: BRAZILIAN CONTEXT

**DOI:** 10.1093/geroni/igad104.0968

**Published:** 2023-12-21

## Abstract

Older adults represent most users in the Brazilian Unified Health System (SUS), which provides at least one service to 57.7% of the national older population. Despite Brazilian legislation ensuring the rights of older adults, health inequities are evident, including barriers to access prevention services, weaknesses and inequities in the distribution of human resources trained to meet the needs of older adults, and several vulnerabilities to the social determinants of health that undermine healthy aging prospects. Family members provide most care, and informal caregivers are neither paid nor receive financial support. The proportion of older adults living with disability has grown significantly over the last decade, and approximately two-thirds of the care-dependent population in Brazil are adults over 65 years of age. Institutional long-term care (LTC) represents a growing part of the Brazilian older population, but several factors challenge the implementation of person-centered care (PCC) in residential LTC. Although 73% of Brazilian families are covered by primary care provided at SUS, only 56.3% of older people in Brazil are registered in the Family Health Strategy, and most people living in LTC facilities do not have access to primary care. The purpose of this presentation is to contextualize how the dissemination and implementation of a PCC culture and the definition of a national conceptual perspective are affected by the immaturity of the Brazilian LTC sector, the lack of coordinated long-term care policies, and ageism in the healthcare sector.

